# Awake breast cancer surgery: strategy in the beginning of COVID-19 emergency

**DOI:** 10.1007/s12282-020-01137-5

**Published:** 2020-07-30

**Authors:** Gianluca Vanni, Marco Pellicciaro, Marco Materazzo, Mario Dauri, Rolando Maria D’angelillo, Chiara Buonomo, Adriano De Majo, Chiara Pistolese, Ilaria Portarena, Alessandro Mauriello, Francesca Servadei, Erica Giacobbi, Agostino Chiaravalloti, Oreste Claudio Buonomo

**Affiliations:** 1 Breast Unit, Department of Surgical Science, PTV Policlinico Tor Vergata University, Viale Oxford 81, 00133 Rome, RM Italy; 2 Department of Emergency and Admission, Critical Care Medicine, Pain Medicine and Anesthetic Science, Policlinico Tor Vergata University, Viale Oxford 81, 00133 Rome, Italy; 3 Radiotherapy Unit, Department of Oncology and Hematology, Policlinico Tor Vergata University, Viale Oxford 81, 00133 Rome, RM Italy; 4 Department of Diagnostic Imaging and Interventional Radiology, Molecular Imaging and Radiotherapy, Policlinico Tor Vergata University, Viale Oxford 81, 00133 Rome, RM Italy; 5 Department of Oncology, Policlinico Tor Vergata University, Viale Oxford 81, 00133 Rome, RM Italy; 6 Anatomic Pathology, Department of Experimental Medicine, Policlinico Tor Vergata University, Viale Oxford 81, 00133 Rome, RM Italy; 7 Department of Biomedicine and Prevention, Policlinico Tor Vergata University, Viale Oxford 81, 00133 Rome, RM Italy; 8 IRCCS Neuromed, UOC Medicina Nucleare, Via Atinense 18, 86077 Pozzilli, IS Italy

**Keywords:** COVID-19, Breast cancer, Awake surgery, Conservative surgery

## Abstract

**Introduction:**

COVID-19 is a declared worldwide pandemic. In our country, due to shortage of hospitals and beds in intensive care unit, oncological and breast cancer (BC) resources are temporarily shifted to COVID-19 patients. In addition, risk of cross-infections should be considered in these frail patients. To accomplish more surgical procedures and to reduce the length of hospital stay (LOS), fast track awake BC surgery should be implemented. The aim of the study is to estimate the effects of surgical shift in our facility during the early COVID-19 outbreak.

**Materials and methods:**

From 30th January 2020 to 30th of March 2020, 86 consecutive patients were retrospectively enrolled and divided into pre-COVID-19-BC and COVID-19-BC. Clinical parameters and anamnestic data were collected and analyzed. Surgical procedures, relative complications and type of anaesthesia were reported. The effect on surgical time (ST), operative room time (ORT) and length of stay (LOS) were described and examined.

**Results:**

No statistical difference was found in complications rate, clinical data and surgical procedures (*p* > 0.05). Awake breast conservative surgery (BCS) was the most frequent procedure in COVID-19-BC (*p* = 0.006). A statistically significant decrease in ORT and LOS was reported in COVID-19-BC (*p* = 0.040 and *p* = 0.0015 respectively), while comparable time resulted for ST (*p* = 0.976). Mean ORT and LOS reduction were 20.79 min and 0.57 hospital bed days.

**Conclusion:**

In the “COVID-19 era”, fast track awake breast surgery provides a reduction of ORT, LOS and potentially surgical treatment for a wider number of oncological patients.

## Introduction

Pandemic coronavirus (COVID-19) caused by severe acute respiratory syndrome COVID-19 (SARS-COV-2), has currently a tremendous impact on Public Health policy as well as in the daily routine of million people worldwide [1]. Western European countries are experiencing more and more the need of hospital and intensive care unit (ICU) beds. Planned construction of temporary COVID-19 hospitals may be not sufficient to control the epidemic during the highest peak. Despite all these measures, rearrangement of operating room into makeshift ICU is progressively taking hold in hospitals [1].

Due to this reason, Italian College of Anaesthesiologists and several oncologic Chinese scientific committees issued some recommendations to face COVID-19 for prevention, risk reduction and correct usage of resources. Even in western country, a coordinated action of physicians’ scientific committee could be able to reduce COVID-19 infection [2–7].

Temporary reallocation of resource towards COVID-19 could significantly have an impact on cancer management. Oncological treatments delay until the end of the outbreak is not feasible for cancer patients, considering also the psychological impact that this may induce. Depression and anxiety experienced during cancer could be enhanced during the pandemic and might impair treatment’s choice [8]. Moreover, cancer patients in the COVID-19 era have higher risk of disease and worse clinical outcome compared to general population [9]. Due to the mentioned issues, COVID-19 outbreaks could result in a forced treatment delay as well as an increased risk of COVID-19 severe outcomes for cancer patients. To decrease cross-infection risk, physicians should evaluate every kind of extraordinary measures.

During this outbreak, many medical center are turning into COVID-19-hospital. In this transition phase, both COVID-19 and oncological patients coexist in the same health service. Despite different ward and compete separated paths, healthcare workers may have difficulty in managing this coexistence. We focused our health policy to both optimize scarce resource and to reduce cross-infection risk as much as possible. In view of our past experience with awake surgeries, we speculated that the past adopted strategy may be helpful during this outbreak. In the previous analysis, we have described the role of the awake surgery in avoiding immune system impairment. Beyond the immunological advantages, this strategy could reduce LOS and respective cross-infection risk between patients, leading at the same time to resources saving.

To maximize the number of surgical procedures and reduce the risk of infections during the early period of COVID-19, we decided to perform the highest percentage of awake breast surgery and fast track surgery when feasible to reduce hospitalization.

The design of our study derives from this assumption and our aim is to estimate how the surgical strategy changed in our facility during the COVID-19 outbreak. Description of control measures taken in the hospital and nationwide to reduce the spread of COVID-19 is beyond the aim of this study and all suggested measures are displayed to describe our findings.

## Materials and methods

### Study design

In this retrospective monocentric study, we evaluated the role of COVID-19 in BC surgical practice change in the Tor Vergata University Hospital. Institutional review of our Department waived the need for a formal approval due to the retrospective nature of the design and due to COVID-19 emergency. All patients who underwent breast surgery from 30th January 2020 to 30th of March 2020 were retrospectively enrolled in the study.

### Population

86 consecutive breast patients treated breast surgery were analysed in the study. From this population, patients were grouped according to surgical period into pre-COVID-19 and COVID-19 patients. 1st March 2020 was defined as the cut-off, meaning the moment when the first not-imported COVID-19 case was registered in Rome. Mean age was 64.77 ± 8.4. Main exclusion criteria were male sex, pregnancy, pure breast reconstruction (BR) surgical procedure. During COVID-19 interval, no contralateral immediate symmetrization was performed and to reduce *bias,* patient who underwent this procedure in the previous period were excluded to the study.

Prior to the first visit, all our patients routinely signed informed consent for data analysis in our clinical practice.

### Data collection

Age, body mass index (BMI), family history or personal history of BC, prior administration of Neoadjuvant chemotherapy (NAC) and length of stay (LOS) data were retrospectively collected from clinical notes. LOS was considered in days and discharge criteria are reported in Table 1. Reoperation was considered when a second surgery was performed within 3 months from the first procedure.

**Table 1:** Discharge criteria

Discharge criteria
Stable vital signs
Alert and orientated
Absence of respiratory distress
Pain controlled
No bleeding (drainage < 100 cc 24 h)
Steady gait, no dizziness or meets preoperative level

Patients that fulfil all these criteria were discharged home

Data obtained from imaging review were used to define clinical staging based on recommendations from AJCC 2018 (edition VIII) of TMN classification. Due to the small size sample, clinical stage was grouped as dichotomous variable as early breast cancer (EBC) or local advance breast cancer (LABC) according to NCCN guidelines [10].

Surgery procedure was distinguished between breast conservative surgery (BCS) and mastectomy (Mx). BCS included all procedures with partial gland removal and Mx comprised the complete removal of the glandular tissue with or without sparing nipple areola complex. All Mx underwent immediate BR using tissue expander or breast implant according to our common clinical practice (NCCN guidelines) [10].

The axillary procedure was analysed in the population. Patients without clinical or radiological lymph nodes involvement underwent sentinel lymph node biopsy (SLNB). Otherwise patients with axillary involvement underwent axillary lymph node dissection (ALND). In addition to SNLB, complementary lymph node removal ≤ 3 lymph node was classified as (SLNB), otherwise it was described as ALND. SLNB patients underwent sentinel frozen section evaluation during the surgical procedure according to NCCN guidelines [10].

Anaesthesiologist strategy was reported as not awake breast surgery (not awake) and awake breast surgery (awake). In the former group, patients were enlisted when supraglottic or subglottic devices were used. Instead, all procedures with regional anaesthesia (RA) (e.g. peripheral nerve block, central neuraxial blocks) or local anaesthetics administration without mechanical ventilation were defined as awake.

Surgical time (ST) was considered as skin to skin surgical time and reported in minutes. Moreover, operating room time (ORT) was defined as from entrance to exit in the surgical area and contained the time of surgical procedure, of surgical theatre preparation and recovery.

Data from surgical specimen were included in the study, when not available due to the short surgical period data from preoperative biopsy as core needle biopsy (CNB) or vacuum assisted biopsy (VAB) were utilized [11, 12]. Tumour maximum diameter was collected and reported in cm. Estrogen receptor (ER), progesterone receptor (PR) and Ki67 index were expressed as percentage of positive cells in specimen studied through immunohistochemistry (IHC). Overexpression of Her2 gene (HER2 +) was identified by IHC or by FISH, as indicated by the recommendations of the 2018 ASCO/CAP and reported as dichotomous variable (HER + yes/no). All patients were classified into the subgroups in accordance with the intrinsic subtypes recommended by 2017 San Gallen International Expert Consensus Report. Due to the small size sample, clinical intrinsic subgroups were described as binary variable: luminal (LUM) and non-luminal (NLUM). LUM group consists of Luminal A, Luminal B + , Luminal B− patients and NLUM of Her2 type and Triple Negative, respectively.

Data from follow up visits and clinical records during hospitalization were evaluated to highlight early complication that was enlisted in our study. Breast-modified Clavien–Dindo classification was applied [13, 14]. Only the complications rated as ≥ 2 according this scale were analysed in the study.

### Statistical analysis

All data were codified into the EXCEL database (Microsoft, Washington, DC, USA). For continuous variables we calculated means and ranges. *T* test was used to determine if there were significant differences between the two groups. Categorical data were recoded into numbers and percentages. Analysis was performed using the Fisher’s exact test. Different surgical procedures were classified as dichotomous variable (e.g. BCT awake + SLNB Y/N) and analyzed with fisher exact test. Variables with assigned *p* values < 0.05 were considered statistically significant. All the statistical analysis was performed in SPSS statistical package version 23.0 (SPSS Inc., Chicago, IL, USA).

## Results

### Clinical data

From 30th January 2020 to 30th of March 2020, we enrolled retrospectively 86 patients in the study. Until 1st of March 39, patients underwent BC surgery (pre-COVID-19-BC group) and 37 cases after the cut-off data (COVID-19-BC group). No statistically significant difference was found in age, BMI, family and personal history of BC. Also, prior NAC administration and surgical reoperation were comparable between the two groups (*p* > 0.05). Mean and relative p value of the above parameters is resumed in Table 2.

**Table 2:** Demographics: age (years), BMI (Kg/m^2^)

	Pre-COVID-19-BC (*n* = 39)	COVID-19-BC (*n* = 37)	*p* value
Age years	64.26 ± 8.67	61.2 ± 7.88	0.428
BMI	22.84 ± 5.04	22.40 ± 5.13	0.747
Family history of BC (%)	11(28.2%)	7 (18.91%)	0.422
Personal history of BC (%)	1 (2.56%)	0 (0%)	1.000
Neoadjuvant chemotherapy	5 (12.82%)	7 (18.91%)	0.539
Reoperation	3 (7.69%)	4 (10.81%)	1.000

Rate of family and personal history of breast cancer, neoadjuvant chemotherapy and reoperation in the Pre-COVID-19-BC and COVID-19-BC group with relative *p*

Clinical presentation showed no statistically significant difference, maximum diameter and EBC rate was similar between the two groups (*p* > 0.05). Also, molecular subtype grouping did not demonstrate statistically significant difference. Analyzing biomolecular expression of BC prognostic and predictive factors (ER, PR, KI67, HER2 Score) values did not reach statistically significant difference between groups in any parameters (*p* > 0.05) (Table 3). Waiting list between BC diagnosis and surgical procedure did not show a statistically significant difference (*p* > 0.05) (11.8 days in pre-COVID-19-BC group and 12.2 days in COVID-19-BC group).

**Table 3:** Tumour diameter in cm, rate of type of clinical presentation in the Pre-COVID-19-BC and COVID-19-BC group with relative *p*

	Pre-COVID-19-BC (*n* = 39)	COVID-19-BC (*n* = 37)	*p* value
Diameter cm (min.-max)	1.98 ± 1.14	2.3 ± 1.71	0.328
Early BC	32 (82.05%)	28 (75.67%)	
Local advanced BC	7 (17.96%)	9 (24.33%)	0.579
Luminal	31 (79.48%)	25 (67.56%)	0.300
Non-luminal	4 (10.26%)	5 (13.51%)	0.733
Missing data	4 (10.26%)	7 (18.93%)	0.340

### Surgical procedure

Analysing surgical procedure, BCS rate was similar between pre-COVID-19-BC and COVID-19-BC groups *(*p > 0.05). Also, axillary management did not show any statistically significant difference between the two periods in analysis (*p* > 0.05). In the COVID-19 period, according to our policy, a number of awake surgery (Table 4) procedures were higher: 73% vs 36% in the control group (*p* = 0.001).

**Table 4:** Type of breast and axillary surgical procedure and anaesthesia management in the Pre-COVID-19-BC and COVID-19-BC group with relative rate and *p*

	Pre-COVID-19-BC (*n* = 39)	COVID-19-BC (*n* = 37)	*p* value
Breast treatment			
Conservative treatment	29 (74.35%)	29 (78.37%)	0.7896
Mastectomy	10 (25.65%)	8 (21.67%)	0.7896
Axillary treatment			
SLNB	34 (87.17%)	33 (89.19%)	1.000
ALND	5 (12.83%)	4 (10.81%)	1.000
Anaesthesia			
Awake	14 (35.89%)	27 (72.97%)	0.001
Non-awake	25 (64.11%)	10 (27.03%)	0.001

### Surgical time procedure

In line with the primary aim of our study, ORT and ST were evaluated. In pre-COVID-19-BC group, ORT and ST were 159.92 ± 43.02 min and 86.02 ± 32.02 min. When compared to COVID-19-BC group, a decrease of ORT was reported (139.13 ± 43.84 min), while comparable time resulted for ST (85.81 ± 31.17 min). Fisher’s exact test showed a significant difference in ORT comparison (*p* = 0.040) whereas ST comparison was *p* = 0.976.

Mean LOS for pre-COVID-19-BC and COVID-19-BC groups were 1.92 ± 1.31 days and 1.35 ± 0.68, respectively. LOS demonstrated a mean reduction 0.57 days between COVID-19-BC and pre-COVID-19-BC showing a statistically significant difference (*p* = 0.015) (Table 5).

**Table 5:** Mean of surgical time (in minutes), operative room time (in minutes), length of stay (days) and surgical complication rate in the Pre-COVID-19-BC and COVID-19-BC group with relative *p*

	Pre-COVID-19 (*n* = 39)	COVID-19 (*n* = 37)	*p* value
Surgical time	86.02 ± 32.02	85.81 ± 31.17	0.976
Operative room time	159.92 ± 43.02	139.13 ± 43.84	0.040
Length of stay (days)	1.92 ± 1.31	1.35 ± 0.68	0.015
Surgical complication (≥ 2 Clavien–Dindo)	2 (5.12%) 2 Clavien II	1 (2.70%) 1 Clavien IIIa	1.000

Surgical complication analyzed did not show any statistically difference (*p* = 1.000). pre-COVID-19 and COVID-19 groups experienced two and one modified Clavien–Dindo ≥ 2 complication, respectively. In pre-COVID-19-BC group, complications were one seroma and one case of postoperative mild anaemia, conservatively treated (Clavien–Dindo II). In COVID-19 group, one seroma occurred successively requiring fine needle aspiration (Clavien–Dindo IIIa).

Finally, different surgical procedure rate according to anaesthesia administered was analyzed. In both study groups, the most common awake surgery was BCS + SLNB and no difference in distribution was reported in matched subtype procedure as resumed in Table 6 (p > 0,05). Despite similar distribution, in the COVID-19-BC group, BCS + SLNB showed a statistically significant difference when compared with pre-COVID-19 groups (67.56% vs 35.89%) (*p* = 0.006). Besides study period, NO ALND was performed under awake surgery (pre-COVID-19-BC vs COVID-19-BC). At Fischer’s exact test on 6 × 2 table comparing surgical procedure in the pre-COVID-19-BC and COVID-19-BC groups *p* = 0.008 (Table 6).

**Table 6:** Type of breast and axillary surgical procedure with relative anaesthesia management and operative room time (minutes) in the Pre-COVID-19-BC and COVID-19-BC groups analyzed with Fisher’s exact test: *p* = 0.008

	Operative room time mean (min)	Pre-COVID-19-BC (*n* = 39)	COVID-19-BC (*n* = 37)
BCS awake SLNB	108.7	14 (35.89%)	25 (67.56%)
BCS no-awake SLNB	155.5	14 (35.89%)	3 (8.10%)
BCS awake ALND	n.a	0	0
BCS no-awake ALND	202.4	1 (2.65%)	1 (2.70%)
MX awake SLNB	197.7	0	2 (5.44%)
MX no-awake SLNB	204.1	6 (15.32%)	3 (8.10%)
MX awake ALND	n.a	0	0
MX non-awake ALND	240.6	4 (10.25%)	3 (8.10%)

In COVID-19-era, we performed two cases (5.44%) of MX associated with immediate prepectoral implant-based breast reconstruction. Differently in the pre-COVID-19-BC, no cases of awake major surgical BC procedure were performed.

## Discussion

Due to COVID-19 outbreak, resource allocations are commonly shifted from elective/semi elective treatments to meet the need of critically COVID-19 patients [1]. To reduce cross-infections and oncological under-treatments, Chinese specialists tried to underline the correct management of gastrointestinal cancers, hepatobiliary cancers and BC during local outbreak [3–7].

For BC, several authors suggested practical individual strategies to reduce risk of COVID-19 disease. Moreover, authors endorsed a reduction of hospital admission policy with enhancement of remote evaluation, usage of depot formulation for drugs and delay of non-urgent medical therapy [4, 7].

Other measures suggested by Chinese guidelines seem to be more difficult to apply in western countries. A clear example is the reduction of movement obliging the choice of hospital facility according to house proximity instead of patients’ free choice [7].

BC Multidisciplinary Team discussion (MDT) should be even more encouraged to decide the best treatment according to resources availability. In addition, during the outbreak, NAC should be strongly recommended to reduce the delay of treatment. Moreover, physicians could take advantage of these schedules as a temporary bridge treatment as well as a chance to treat patient with BC subset with low rate of pathological complete response, as LUM patients [15, 16]. In this subset of patients, MDT could consider the partial response or the stable disease after neoadjuvant therapy as affordable goals during local outbreak peak to delay surgery and reduce risk of contamination. In our analysis, we do not demonstrate any statistical difference in surgical patients with previous NAC. We expect these data due to study period (early outbreak of COVID-19). Our hypothesis is that the rate of surgical treatment after NAC will rise in a long time period.

In our facility, surgical policy changed during COVID-19 outbreak and BR surgery was delayed to reduce infections risk during peaks. Moreover, we decided not to perform pure BR to shift all of our residual resource facilities toward oncological patients.

According to our resource saving policy, our surgical strategy was planned to reduce as much as possible invasive surgery, choosing awake surgery and (Enhanced Recovery after Surgery) ERAS protocols. Resource saving policy could lead to more operative room and beds availability, even for COVID-19 patients. This way, BCS could not be restricted to frail patients only as general practice [17, 18], but extended to other patients. When Mx is not avoidable, breast reconstruction choice should be made by surgeon to reduce as much as possible the surgical stress and to allow fast discharge at home, as a classical paradigm [19]. Pre-pectoral tissue expander and breast implant should be preferred to reduce pain and operation time [20, 21]. However, in our series, surgical strategy distribution did not change between BCS and Mx. In our opinion, the choice of surgical procedure of primary tumor was mostly affected by well-known variables such as breast volume, clinical tumor size and location of the tumor [22]. Similarly, axillary management was not affected by the period of treatment. Moreover, the similar rate of surgical procedures during the two periods reduces the risk of possible enrolment bias in our analysis.

Based on our previous experience in awake-BCS, we speculated this strategy during the outbreak. In the previous analysis, we have demonstrated a significant reduction of immune system impairment [23–25].

In COVID-19 group, as showed in our analysis, we pushed this strategy of treatment. On one hand, the immunological advantages associated with a reduction of time exposure could reduce cross-infection risk. On the other hand, achieving a reduction in operative room and hospital beds occupation could lead to resource saving.

In COVID-19-BC group, two patients underwent to awake MX. Both patients had an uneventful postoperative period and were discharged home on the first operative day. Due to small number of awake major BC surgery, further prospective studies are needed to evaluate feasibility as well as clinical and immunological advantages of this strategy.

Awake breast surgery plus regional anaesthesia (RA) provide the possibility of a day surgical management in more and more cases [26, 27], in addition, non-intubated surgery with patients with PPE (personal protective equipment’s) reduces the risk of cross contamination by health providers during invasive procedures [28]. Benefits of RA consist on preservation of respiratory function and airway protective mechanisms, avoidance of aerosolization and hence viral transmission [28]. During 2003 SARS outbreak, intubation and O2 administration resulted as an independent factor for super-spreading nosocomial outbreaks affecting healthcare workers [29]. Proper administration of sedation and oxygen therapy should be taken into account during RA. In case of face mask, low level of oxygen delivery should be maintained to reduce the dispersal distance of exhaled air, as described by Hui et al. [30]. Indeed, different rate of anesthesiologic approach was found in the pre-COVID-19 and COVID-19 groups. Higher rate of awake surgery in the COVID-19 period permitted a statistically relevant reduction in LOS (1.35 vs 1.92). In our experience, awake surgery did not altered OT which is affected mostly by surgical expertise and SLNB frozen section. Conversely, a higher rate of awake surgical procedure was linked to a statistically significant reduction of ORT. ORT, as mentioned before, represents all the time spent in the surgical room by patients and ORT reduction in COVID-19 period could eventually result in a reduced risk of cross-infection in places like recovery room with high patients’ turnover.

From our data, we found that axillary procedure was the factor that influenced more the type of anaesthesia. ALND were always performed under general anaesthesia both in BCS and MX.

Despite ERAS protocol has been shown to improve outcomes in intermediate-to-high-risk surgery, its role in the low-risk surgery has not been fully studied. Ackerman et al. demonstrated a statistical different in reduction of LOS, increase of hospital surgical bed days and consequently surgical volume after implementation of ERAS protocol for mastectomy [31]. Moreover, evidence in literature shows how breast cancer surgery performed without the requirement for hospitalization could be a feasible option for selected patients [32]. In COVID-19 period, mean ORT reduction was 20.79 min with a statistical significant difference. Despite less resources and operative room availability during the early outbreak in our surgical practice, we performed a comparable amount of BC patients without altering our oncological waiting list. Enhancement of fast track awake surgery provided 18.15 more hours (1.65 operatory room day) of surgery room availability. During the outbreak, these saved resources have been reallocated to other oncological procedures.

ERAS awake fast track surgery demonstrated a significative reduction of LOS. Mean reduction was 0.57 days for each patient, so reducing cross-infection risk. Moreover, fast track surgery permitted faster patients turnover, and more availability of hospital beds that can be reallocated according to the need. When compared with control period with this measure, hospital gains 21.09 hospital beds day during COVID-19 interval.

Retrospective and single study institution design with a small size sample could be a limitation for the analysis, but feasibility of a prospective, multicentric study in this outbreak period should be considered. Moreover, due to the short follow up, it is not possible to underline how these measures affected COVID-19 spread in our Institution, disease recurrence, the rate of long-term complication or BR aesthetic results. Despite the lack of this data, early evidence on surgical management in this period could be useful to support different surgical strategy and to demonstrate further clinical evidence. However, the aim of our study was to assess how our clinical practice changed during the early outbreak of coronavirus and to underline which measures could be implemented to improve and accelerate recovery after surgery thus reducing healthcare costs [33, 34]. Another limitation of our analysis was the absence of data regarding immunological impact of different surgical strategies among this population. To avoid any detrimental effect on our patients during this period, all our prospective study on postoperative immune response was temporarily interrupted in our institution. For this reason, these data were not included in the analysis [23–25]. Allocate properly our facility resources to maximize the number of patients treated during surgery room shortage is mandatory to avoid delay and any risk for oncological patients.

During COVID-19 outbreak, surgeon program should take into account the peculiar characteristic of the period. Correct management of residual resources is a necessary ethical conduit toward BC patients and all patients. Awake breast surgery could be a real solution. The reduction of ORT and LOS should be strongly encouraged to maintain as much as possible oncological surgical activity and provide surgical treatment for a wider number of oncological patients. Psychological aspect should be considered in this analysis; fast discharge is also widely accepted by patients in this period due COVID-19-related fear. Moreover, the COVID-19 crisis could be a chance for many breast centers to obtain local evidence to implement these learnings to apply in future normal circumstances. In particular, in our clinical setting, this preliminary finding will help to underline how outpatient surgical program will eventually be planned in the future. Further study should assess the role of ERAS protocol in reduction of COVID-19 spread.
